# Nucleation and Stability of Toron Chains in Non-Centrosymmetric Magnetic Nanowires

**DOI:** 10.3390/nano13121816

**Published:** 2023-06-07

**Authors:** Sebastián Castillo-Sepúlveda, Rosa M. Corona, Eduardo Saavedra, David Laroze, Alvaro P. Espejo, Vagson L. Carvalho-Santos, Dora Altbir

**Affiliations:** 1 Grupo de Investigación en Física Aplicada, Facultad de Ingeniería, Universidad Autónoma de Chile, Avenida Pedro de Valdivia 425, Providencia 7500912, Chile; sebastian.castillo@uautonoma.cl; 2 Departamento de Física, CEDENNA, Universidad de Santiago de Chile, Avenida Víctor Jara 3493, Estación Central, Santiago 9170022, Chile; rosa.corona@usach.cl (R.M.C.); a.p.espejo@gmail.com (A.P.E.); 3 Department of Physics, University of Santiago de Chile (USACH), Santiago 9170124, Chile; eduardo.saavedra.d@usach.cl; 4 Instituto de Alta Investigación, Universidad de Tarapacá, Casilla 7D, Arica 1000000, Chile; dlarozen@uta.cl; 5 Departamento de Física, Universidade Federal de Viçosa, Avenida Peter Henry Rolfs s/n, Viçosa 36570-000, MG, Brazil; vagson.santos@ufv.br

**Keywords:** torons, metastable states, micromagnetism, Bloch points

## Abstract

This work analyzes the magnetic configurations of cylindrical nanowires with a bulk Dzyaloshinskii–Moriya interaction and easy-plane anisotropy. We show that this system allows the nucleation of a metastable toron chain even when no out-of-plane anisotropy exists in the nanowire’s top and bottom surfaces, as usually required. The number of nucleated torons depends on the nanowire length and the strength of an external magnetic field applied to the system. The size of each toron depends on the fundamental magnetic interactions and can be controlled by external stimuli, allowing the use of these magnetic textures as information carriers or nano-oscillator elements. Our results evidence that the topology and structure of the torons yield a wide variety of behaviors, revealing the complex nature of these topological textures, which should present an exciting interaction dynamic, depending on the initial conditions.

## 1. Introduction

Recent advances in experimental techniques for the fabrication of magnetic nanostructures have promoted nanomagnetism as a topic of intense research from both applied and fundamental points of view. One of the main appealing properties of nanomagnets is the possibility of nucleating magnetization textures that behave as single particles that can be controlled by means of electric currents, magnetic fields, and temperature gradients. Thus, an in-depth analysis of the behavior of these quasiparticles is crucial for using them as information carriers in spintronic-based devices [1,2,3]. Initially, due to experimental limitations for fabricating nanostructures, one of the main focuses of the research in nanomagnetism was the static and dynamical properties of one or two-dimensional magnetic quasiparticles, such as domain walls [4,5], vortices [6,7,8], and skyrmions [9]. Nevertheless, technological advances have made the fabrication of three-dimensional nanomagnets possible [10,11,12,13], yielding the emergence of a new and challenging research area—three-dimensional nanomagnetism [14,15,16].

An exciting feature associated with the inclusion of the third dimension in nanomagnetism is the appearance of novel magnetic quasiparticles that share the symmetries of the nanostructure geometry [17,18,19,20,21,22,23,24,25]. Some of these magnetic textures present topological protection, ensuring strong stability even if the magnetization profile is not the groundstate. Among such three-dimensional textures, we highlight hopfions [25,26,27,28,29,30,31], skyrmion strings [32,33], and Bloch points (BPs) [34,35,36]. In particular, the latter has been predicted, for instance, in cylindrical nanowires [36,37,38,39] and consist of magnetic textures in which, in a closed surface around its center, the magnetization field covers the solid angle an integer number of times, defining the BP topological charge [40,41,42,43]. The nucleation and stabilization of several BPs in non-confined systems have been previously reported. For instance, due to the screw dislocation of the conical phase in cubic chiral magnets, a magnetic state consisting of an alternating core magnetization separated by BPs [44] is obtained. These results were also corroborated by Monte Carlo simulations, revealing that a pair of coupled BPs may appear as a stable state in cubic chiral magnets [45]. Okumura et al. [46] have also shown the possibility of nucleating BP lattices from an effective spin model with long-range interactions arising from the itinerant nature of electrons. Finally, the experimental observation of BP lattices in chiral magnets through high-resolution Lorentz transmission electron microscopes has been reported by Tanigaki et al. [47].

The presence of two or more BPs in a nanomagnet can create torons, which are magnetic textures consisting of a skyrmion tube whose ends collapse into two opposite singularities [48,49]. It has been predicted that one magnetic toron can stabilize in chiral cylindrical nanodots with vanishing anisotropy sandwiched between two layers of a material with high perpendicular magnetic anisotropy [50,51,52]. In this case, the strong anisotropy at the top and bottom surfaces of the nanowire favors the spin alignment parallel to the nanodot axis, allowing the nucleation of one toron confined between two BPs. Nevertheless, if the nanodot top and bottom layers do not present perpendicular magnetic anisotropy, the BPs are annihilated, the toron loses its stability, and a skyrmion tube nucleates [52]. In addition, Hu et al. showed that the stabilization of a toron in a cylindrical nanowire with a bulk Dzyaloshinskii–Moriya interaction (DMI) can be achieved by applying a magnetic field parallel to the nanowire axis [53].

Based on the above, the nucleation and stabilization of torons is an open topic that is currently receiving great attention. Therefore, this work analyzes the magnetic configurations of cylindrical nanowires with bulk DMI and intrinsic easy-plane anisotropy through micromagnetic simulations. Our results show that a certain number of torons can be nucleated, creating a metastable toron chain. The number of nucleated torons depends on the nanowire length and can be manipulated through a uniaxial magnetic field.

## 2. Theoretical Model

**Figure 1:**
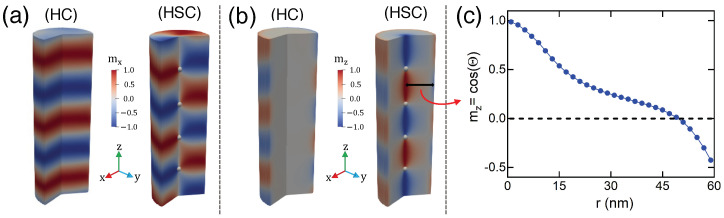
Magnetization components pointing along the directions *x* (**a**) and *z* (**b**) for the HC (left) and HSC (right). Panel (**c**) depicts the *r*-component of the magnetization profile along the cross-section represented by the black line in (**b**), located in the median plane between two BPs.

In this work, we adopt the micromagnetic approach to analyze the magnetic properties of the nanostructure. Therefore, the magnetic body is assumed to be a continuum material where the magnetization vector has the same magnitude throughout but can vary in direction from point to point. In this approach, all information about the lattice type is included in the material parameters [54]. We start by considering a cylindrical magnetic nanowire with radius *R* and length *L*. To obtain different three-dimensional magnetic textures as a function of the nanowire length, we fixed R=60 nm and consider that *L* ranges from 20 nm to 200 nm. The magnetic energy, *E*, is given by the sum of the exchange, dipolar, bulk DMI, and easy-plane anisotropy in the xy-plane (see Figure 1a).

To obtain the relaxed states, we perform micromagnetic simulations using the Mumax3 [55] code, a numerical time integration of the Landau–Lifshitz–Gilbert equation [56,57]:

(1) $$\frac{d\vec{m}}{dt}=-\gamma \vec{m}\times {\vec{H}}_{\mathrm{eff}}+\alpha \left(\vec{m}\times \frac{d\vec{m}}{dt}\right)\;,$$

where γ is the gyromagnetic ratio, ${\vec{H}}_{\mathrm{eff}}=-\left(1/\mu_{0}M_{s}\right)\delta E/\delta \vec{m}$ is the effective field, and α is a dimensionless dissipation parameter. For the simulations, the system is discretized using a small mesh size of 2×2×1 nm^3^, with a damping α=0.5. Additional calculations were performed with 5×5×5 nm^3^ and 2×2×2 nm^3^ cells, but no appreciable changes were observed. To obtain the final state, the Landau–Lifshitz–Gilbert equation was integrated on the whole system until the torque of the system reached $10^{-4}$ T. Once the above torque convergence criterion was reached, the magnetization and energy densities were obtained. For all our calculations, we consider that the material parameters are an exchange stiffness $A=8.78\times 10^{-12}$ J/m, a saturation magnetization $M_{s}=3.84\times 10^{5}$ J/m^3^, and a DMI constant $D_{b}=1.58\times 10^{-3}$ J/m^2^. These values are in the order of magnitude of the parameters characterizing several magnetic materials, such as FeGe [58] and MnSi [59].

Previous works showed that the ground state of isotropic chiral magnetic nanowires consists of skyrmion tubes, helical, or conical states [60], and no torons were reported in such systems. Therefore, to enable the nucleation of torons in the nanowires, we consider a material with an easy-plane anisotropy (xy-plane) characterized by a magnitude $K=0.14\times 10^{6}$ J/m^3^. We highlight that we have also calculated the stable configurations in nanowires with vanishing anisotropies. The wide variety of magnetic excited states reported in [58] has been obtained. Nevertheless, in this work, we do not present these results since we aim to find the conditions for obtaining a toron chain.

## 3. Results

The described model allows us to obtain the magnetization relaxed states as a function of *L* using two initial configurations: a ferromagnetic state and multiple domains with opposite directions. We start by obtaining the relaxed configurations starting from an initial state given by $m_{x}=1$ and with a vanishing magnetic field. Our results reveal that the final magnetic pattern consists of an in-plane helicoidal configuration (HC), with period *P*, as depicted in Figure 1a ($m_{x}$-component) and Figure 1b ($m_{z}$-component). To characterize the HC magnetization field, we parameterize it in a Cartesian basis as ${\vec{m}}_{\mathrm{HC}}=\cos\Theta_{\mathrm{HC}}\hat{z}+\sin\Theta_{\mathrm{HC}}\hat{\upsilon };\;\hat{\upsilon }=\cos\Phi_{\mathrm{HC}}\hat{x}+\sin\Phi_{\mathrm{HC}}\hat{y}$, where $\Theta_{\mathrm{HC}}=\Theta_{\mathrm{HC}}\left(z,\;r\right)$ describes the cross-section magnetization profile and $\Phi_{\mathrm{HC}}\left(z,\;r\right)$ defines the magnetization helicity. Therefore, the HC can be described by

(2) $$\begin{array}{c} \Theta_{\mathrm{HC}}=\frac{\pi }{2}\;,\;\mathrm{and}\;\Phi_{\mathrm{HC}}=\frac{2\pi }{P}\;z\;. \end{array}$$

One can notice that the HC consists of single-domain layers in the xy-plane. The magnetization of one layer is rotated by a certain angle relative to the next layer. The rotation period depends on all magnetic interactions of the system [61]. The period of the HC when exchange and DMI interactions are present was previously predicted [62], giving $4\pi A/D_{b}$, which, for the material parameters used in this work, gives ≈69.8 nm. Our simulations predict 68.3 nm, independent of *L*. The small difference with respect to the period obtained in [62] originates from the inclusion, in our model, of the magnetostatic and anisotropy contributions to the energy.

The next step of our work is to analyze the nucleation of multiple BPs in the nanowires. It has been previously shown that an initial state composed of monodomains with magnetization pointing along opposite directions in successive regions present BPs as relaxed states [39]. We follow this idea and divide the magnetic nanowires into *n* sections along the *z*-axis, with *n* ranging from 1 to 6, each one with a magnetization given by $m_{z_{i}}={\left(-1\right)}^{i}$, where i∈[1, n]. For instance, if the nanowire is divided into three sections, $m_{z_{1}}=-1$, $m_{z_{2}}=1$, and $m_{z_{3}}=-1$. To create this starting antiferromagnetic configuration, it is possible to surround the wire with a multi-layered vertical array [63] of planar or low-profile coils [64,65], connected to a power supply in alternating opposite directions. We then relax the system and obtain final states defined by the initial conditions characterized by *n*. We compared the different energies from the obtained final states, finding a multi-BP state with lower magnetic energy as a function of the nanowire length. Our results show the appearance of configurations whose number of BPs depends on *L*. The region between two BPs exhibits a magnetic pattern similar to an “incomplete skyrmion” that continuously changes its helicity as a function of *z*. Thus, from now on, this state will be called helicoidal skyrmion configuration (HSC). These magnetization configurations are depicted in Figure 1a ($m_{x}$-component) and Figure 1b ($m_{z}$-component), where the white dots represent the position of the nucleated BPs and the colored regions illustrate the component of the magnetization as indicated in the color bar. The magnetization component of the incomplete skyrmion pointing along the xy-plane at the middle between two BPs is presented in Figure 1a,c. It is worth noting that the magnetization in the border of the incomplete skyrmions in the intermediary layers does not reach saturation along the *z*-axis, creating what we can call a “half-skyrmion”.

**Figure 2:**
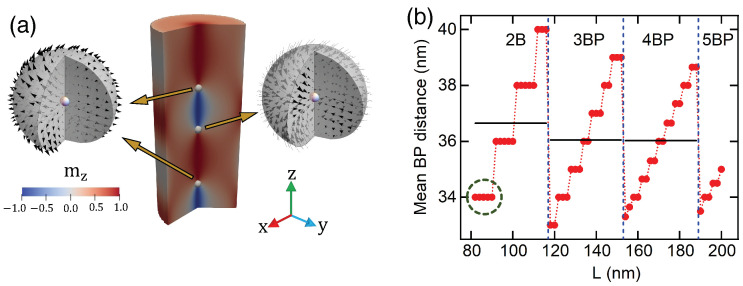
Panel (**a**) presents the final state of the torons, with the respective BP patterns. (**b**) presents the distance between two consecutive BPs as a function of *L*. Each section corresponds to a fixed number of BPs. Black lines represent the average value of *d* in the range of lengths in which a given number of BPs are nucleated.

The magnetization between two consecutive BPs forms a set of monolayers with a chiral magnetic ordering with different helicities. Thus, if we consider a BP with an in-plane magnetization pointing radially outward, the subsequent BP has a magnetic in-plane configuration pointing inward. We can describe the magnetization field near each BP in terms of the polar (ϑ) and azimuthal (φ) angles as $\Theta_{\mathrm{BP}}=p\vartheta +\pi \left(1-p\right)/2$ and $\Phi_{\mathrm{BP}}=q\varphi +\gamma$, where the parameter p=±1 defines the BP polarity, q=±1 is its vorticity, and γ is the helicity. While the polarity defines the direction along the *z*-axis in which the magnetic moments point (outward for p=+1 or inward for p=−1), the vorticity allows us to determine if we have a vortex (q=+1) or an antivortex (q=−1) in the xy-plane. The helicity defines the rotation direction of the vortex in the xy-plane. In this context, the divergent hedgehog-type BP is a vortex Bloch point with positive polarity (p=1, q=1, γ=0), and the convergent hedgehog-type BP is a vortex Bloch point with negative polarity (p=−1, q=1, γ=π). Under this framework, the properties of the BP can be described by the topological Pontryagin invariant [18,66,67], defined as $Q={\left(4\pi \right)}^{-1}\int_{\Omega }\sin\Theta_{\mathrm{BP}}\;d\Theta_{\mathrm{BP}}\;d\Phi_{\mathrm{BP}}=pq$, where Ω is a spherical surface enclosing the BP. It is worth noting that Malozemoff and Slonczewski [43] have classified BPs by using the flux N of the gyrotropic vector, whose value is N=−Q. In this case, two consecutive BPs have the same vorticities but opposite polarities, as shown in Figure 2a. Consequently, two consecutive BPs have opposite topological charges.

As mentioned above, magnetic textures composed of skyrmion tubes between two BPs with opposite polarity and helicity are known as torons. Therefore, the HSC evidences the formation of a certain number of torons as a function of *L*. The number of torons, $n_{\mathrm{HSC}}$, is related to the number of BPs nucleated into the system, $n_{\mathrm{BP}}$, as $n_{t}=n_{\mathrm{BP}}-1$. The adopted parameters allow the nucleation of one BP when L=45 nm. Such a unique BP is a local minimum state until L=80 nm, when two BPs appear, nucleating a first toron. When increasing *L*, a new BP appears for L=115 nm, defining two torons. Every 35 nm, a new BP appears, periodically increasing $n_{t}$. The nucleation of toron chains has been theoretically predicted as screw dislocations in non-confined chiral magnetic systems [44]. In confined systems, it is only possible to obtain torons when a perpendicular magnetic anisotropy in the nanodot upper and lower surfaces is included [50,51,52]. However, the nucleation of more than one toron in confined systems was not previously reported.

To describe the HSC, we adopt a parameterization of the magnetization in a cylindrical coordinate basis as ${\vec{m}}_{\mathrm{HSC}}=\cos\Theta_{\mathrm{HSC}}\hat{z}+\sin\Theta_{\mathrm{HSC}}\hat{\upsilon };\;\hat{\upsilon }=\cos\Phi_{\mathrm{HSC}}\hat{\rho }+\sin\Phi_{\mathrm{HSC}}\hat{\varphi }$. Here, $\Theta_{\mathrm{HSC}}=\Theta_{\mathrm{HSC}}\left(z,\;r\right)$ describes the magnetization profile in each layer, and $\Phi_{\mathrm{HSC}}\left(z,\;r\right)$ defines the helicity of the magnetization pattern. Under this assumption, the HSC can be represented by the ansatz

(3) $$\begin{array}{c} \Theta_{\mathrm{HSC}}=\theta \left(r,\;z\right)\pm \;\frac{\pi }{2}\;\mathrm{and}\;\Phi_{\mathrm{HSC}}=\kappa z\;, \end{array}$$

where θ(r, z) describes deviations from the purely in-plane state in each layer, with θ(0, z)=π/2. The sign ± depends on the magnetization direction in the central line connecting two BPs. Besides, κ gives the period of the HSC, depending on *L* and the material magnetic parameters. An important feature of the obtained metastable states is the total magnetization for an odd or even number of BPs. In this case, if $n_{\mathrm{BP}}$ is even, $\langle M_{z}\rangle \neq 0$, with the nucleation of chiral bobbers [68,69] with the same polarity at the wire ends. In contrast, when $n_{\mathrm{BP}}$ is odd, $\langle M_{z}\rangle \approx 0$, and chiral bobbers with opposite polarities are nucleated at the extremes.

The BP-to-BP distance allows us to determine the toron size as a function of *L*. Figure 2b presents the distance between two consecutive BPs (red dots), and the length at which a new BP is nucleated (blue-dashed lines). The number, *n*, of BPs in each region is denoted as *n*BP. Horizontal black lines illustrate the average value of the toron’s size for each length interval with a fixed $n_{\mathrm{BP}}$. As expected, in each subregion (see the green circle, for example), the BP-to-BP distance is approximately constant while increasing *L*, which is compensated by an increase in the size of the chiral bobbers at the extremes of the cylinder. However, at particular length values (for example, the upper limit of the region enclosed by the green circle), the BP-to-BP distance increases, being compensated by a decrease in the chiral bobber size. When a new BP is nucleated, the toron’s size abruptly diminishes, evidencing a repulsive interaction between BPs. Our results enable us to estimate a limiting value for the toron’s size when L→∞ of approximately 36 nm.

Until now, we have obtained toron chains characterized by half-skyrmions hosted between two BPs. However, by applying uniaxial magnetic fields, it is possible to nucleate toron chains with integer skyrmions. In this context, we consider a magnetic field defined as H=Hz and analyze the change in the properties of the BPs (and torons) as a function of *H*. We consider the HSC as initial state at H=0, turn on a magnetic field, and relax the system until the magnetization stabilizes under this new condition.

**Figure 3:**
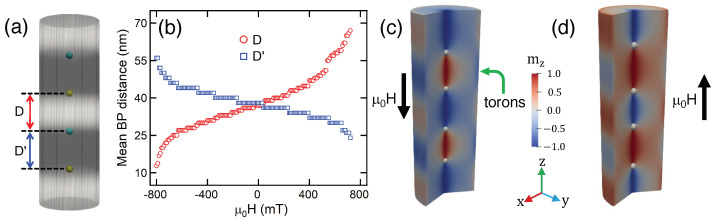
(**a**) Schematic representation of a toron chain under a magnetic field. (**b**) Distance between two BPs separated by a magnetic domain with a magnetization parallel (antiparallel) to the external magnetic field, represented by red (blue) lines. (**c**,**d**) Shrunk and stretched torons and chiral bobbers for magnetic fields pointing in different directions.

Our results show that, depending on the direction of the magnetic field, we can determine that the BPs approach or move away from each other, allowing toron shrinking or stretching. We define *D* ($D^{\prime }$) as the BP-to-BP distance when the magnetic moments between the BPs point upward (downward) (see Figure 3a). Figure 3b illustrates *D* (red) and $D^{\prime }$ (blue) as a function of *H*. As expected, if H<0, $D^{\prime }$ (*D*) increases (decreases) with *H*, while for H>0, the opposite occurs, and $D^{\prime }<D$. The changes in *D* and $D^{\prime }$ allow the nucleation of torons with a complete skyrmion in between, as depicted in Figure 3c (red domains) and Figure 3d (central blue domain). The analysis of these figures reveals that for H<0, blue domains (m pointing downward) increase, shrinking the torons, depicted by a green arrow (red domains in Figure 3c). As expected, the opposite is observed for H>0, and the integer skyrmion is trapped in the middle plane of the blue domain, as shown in Figure 3d.

**Figure 4:**
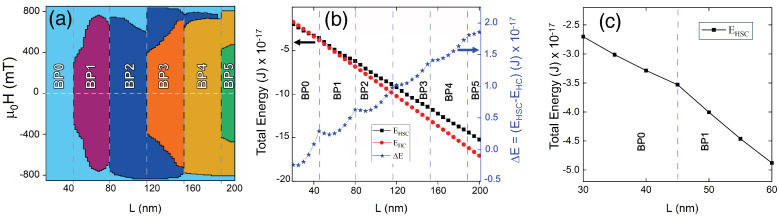
Panel (**a**) presents the state diagram showing $n_{\mathrm{BP}}$ as a function of *L* and *H*. The horizontal white-dashed line denotes H=0. Each colored region corresponds to a different number of BPs. (**b**) Magnetic energy of the HC (red dots) and HSC (black squares), for H=0, and energy difference between these states (blue stars) as a function of *L*. (**c**) Enlarged view of the magnetic energy of the HSC, for H=0.

In addition to the possibility of controlling the number of BPs by varying *L*, we can also use an external magnetic field to change $n_{\mathrm{BP}}$. In this context, Figure 4a presents a state diagram depicting the number of BPs as a function of *L* (in steps of 2 nm) and *H*. This evidences that the $n_{\mathrm{BP}}$ decreases as the magnetic field increases. One can notice that the annihilation field depends on *L* and $n_{\mathrm{BP}}$. The results show that until $|\mu_{0}H|=300$ mT, the torons change their sizes, but $n_{\mathrm{HSC}}$ remains constant, in such a way that if the magnetic field is turned off, the system returns to its original state with the same $n_{\mathrm{HSC}}$ for each *L* value. If the number of BPs is even, the range of magnetic fields for which the state returns to the original one is wider and can reach even $|\mu_{0}H|=600$ mT. Finally, for the range of parameters considered here, we also notice that a magnetic field $|\mu_{0}H|=900$ mT annihilates all the BPs from the nanowire, and the system reaches a single domain state with the magnetization oriented along the magnetic field.

An intriguing behavior observed in Figure 4a is the asymmetry in the annihilation field for an even number of BPs. If the magnetic field is applied along the *z* direction in a system in which *L* is in the range of 100 to 200 nm, the two BPs hosted in the system are annihilated at the same time for $\mu_{0}H=-800$ mT. However, if the magnetic field is applied along the −*z* direction, BPs at respective *L* values are annihilated at $\mu_{0}H=600$ mT. The same phenomenon is observed when the NW length allows the nucleation of four BPs. In this case, for L∈[150, 160] nm, a magnetic field $\mu_{0}H=-800$ mT annihilates all BPs simultaneously. On the other hand, two BPs are annihilated from the system when $\mu_{0}H=600$ mT and the other two at a magnetic field $\mu_{0}H=800$ mT.

**Figure 5:**
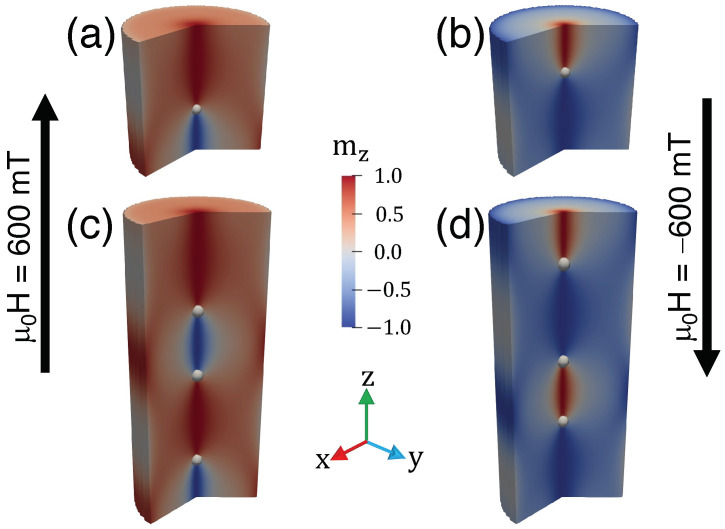
(**a**,**b**) stable chiral bobbers for L=60 nm, and (**c**,**d**) stable chiral bobbers and torons for L=140 nm. The change from $B_{z}\to -B_{z}$ in these magnetic configurations implies two transformations: z→L−z and $m_{z}\to -m_{z}$.

To understand this asymmetry, which does not occur for a nanowire hosting an odd number of BPs, we analyze the magnetic pattern when the magnetic field is applied to systems nucleating an odd or even number of BPs. Results are presented in Figure 3c,d and allow us to conclude that the asymmetry in the annihilation field when an even number of BPs is nucleated comes from the existence of two different annihilation processes, depending on the magnetic field direction. If we apply a magnetic field H>0 into the system, as shown in Figure 3d, the shrinking of one toron occurs, while the two BPs near the wire ends approach the top and bottom surfaces. On the other hand, for H<0, we see the shrinking of the two integer torons, which is followed by the motion of two BPs near the ends to the inside of the nanowire (see Figure 3c). This behavior does not occur when an odd number of BPs nucleates in the nanowire—a behavior that is accompanied by appearance of the same number of torons. In both cases, H>0 and H<0, one of the BPs near the wire end moves toward the nanowire top or bottom surface while the other moves to the inside. The motion of the BPs yields the shrinking and stretching of the same number of torons, giving rise to the same annihilation process independent of the direction of the external magnetic field. This behavior is shown in Figure 5, where we present nanowires hosting one (Figure 5a,b) and three BPs (Figure 5c,d). The asymmetry in the annihilation process of torons points to an interesting hysteretic phenomenon. Because the annihilation field strength depends on the direction of the external magnetic field, there should be a bias in the hysteresis loop coming from the different annihilation processes when an even number of BPs are nucleated in the nanowire.

We have also analyzed the magnetic energy for H=0 of the HC ($E_{\mathrm{HC}}$) and HSC ($E_{\mathrm{HSC}}$) states as a function of the nanowire length. Our results are presented in Figure 4b, evidencing that, although the energy of each configuration decreases as a function of *L*, the HC (red dots) has lower energy than the HSC (black squares) in the interval ranging from L=40 nm to L=200 nm. Therefore, one can conclude that systems with BPs have more energy than HC. Figure 4c includes an enlarged view of the HSC state energy, showing that its behavior as a function of *L* is not a straight line. Because of that, when analyzing the energy difference ($\Delta E=E_{\mathrm{HSC}}-E_{\mathrm{HC}}$) between these states (blue stars), we observe the existence of local minima of ΔE for each number of BPs, corresponding to the length at which a larger stability of the BPs (and consequently of the torons) is achieved, even if $E_{\mathrm{HSC}}>E_{\mathrm{HC}}$. These results are corroborated by numerical simulations that show that, once the BPs are nucleated, they stay in the system for a long time.

## 4. Conclusions

In summary, we analyzed the possibility of nucleating torons in nanowires with a chiral interaction and easy-plane anisotropy. We showed that, depending on the nanowire length, several BPs with periodic helicities appear in the system, yielding the nucleation of a toron chain. We also analyzed the behavior of the considered system under the action of a magnetic field. In this case, the magnetic field shrinks and stretches the observed torons by forcing BPs to approach each other. If the magnetic field is strong enough, BPs are annihilated from the system, and the number of nucleated torons decreases. We obtained a state diagram to determine the number of BPs and torons as a function of the magnetic field. In the range of considered geometrical parameters, all torons are annihilated for a magnetic field of $\mu_{0}H=\left|900\right|$ mT. Finally, we evidenced that asymmetries in the BPs annihilation magnetic fields for systems hosting an even number of BPs are associated with their response to magnetic fields pointing along the *z* or −*z* directions. These results contribute to the advancement of the discussion about the nucleation and annihilation of torons in magnetic systems.

## Data Availability

All data that support this study are included within the article.

## Abbreviations

The following abbreviations are used in this manuscript:

BP	Bloch point
DMI	Dzyaloshinskii–Moriya interaction
HC	Helicoidal configuration
HSC	Helicoidal skyrmion configuration
